# Investigating the impact of feedback update interval on the efficacy of restorative brain–computer interfaces

**DOI:** 10.1098/rsos.170660

**Published:** 2017-08-30

**Authors:** Sam Darvishi, Michael C. Ridding, Brenton Hordacre, Derek Abbott, Mathias Baumert

**Affiliations:** 1 School of Electrical and Electronic Engineering, The University of Adelaide, Australia; 2 The Robinson Research Institute, The University of Adelaide, Australia; 3 School of Health Sciences, University of South Australia, Australia

**Keywords:** brain–machine interface, brain–computer interface, stroke, feedback, feedback update interval, rehabilitation

## Abstract

Restorative brain–computer interfaces (BCIs) have been proposed to enhance stroke rehabilitation. Restorative BCIs are able to close the sensorimotor loop by rewarding motor imagery (MI) with sensory feedback. Despite the promising results from early studies, reaching clinically significant outcomes in a timely fashion is yet to be achieved. This lack of efficacy may be due to suboptimal feedback provision. To the best of our knowledge, the optimal feedback update interval (FUI) during MI remains unexplored. There is evidence that sensory feedback disinhibits the motor cortex. Thus, in this study, we explore how shorter than usual FUIs affect behavioural and neurophysiological measures following BCI training for stroke patients using a single-case proof-of-principle study design. The action research arm test was used as the primary behavioural measure and showed a clinically significant increase (36%) over the course of training. The neurophysiological measures including motor evoked potentials and maximum voluntary contraction showed distinctive changes in early and late phases of BCI training. Thus, this preliminary study may pave the way for running larger studies to further investigate the effect of FUI magnitude on the efficacy of restorative BCIs. It may also elucidate the role of early and late phases of motor learning along the course of BCI training.

## 1. Introduction

According to the World Health Organization, 15 million people suffer stroke each year, where almost one-third thereof do not adequately recover after stroke [1]. One of the major stroke aftermaths is hemiparesis of the upper limb, severely restricting the ability to perform activities of daily living. Rehabilitation of the upper limb motor functions is a key element of traditional stroke therapies. However, stroke rehabilitation techniques such as physiotherapy do not provide sufficient improvement with at least 30% of stroke victims unable to move their affected arms following therapy [2]. To address this gap, application of motor imagery (MI), which is associated with activity in a network similar to that active during actual movement, has been proposed [3,4]. MI offers a unique opportunity for those 30% of stroke patients with no residual hand movement through activation of the perilesional brain areas of the damaged hemisphere. This brain activation if properly coupled with real-time sensory feedback closes the sensorimotor loop [5], and might result in neuroplastic changes that are beneficial for motor recovery following stroke [5,6]. Brain–computer interfaces (BCIs) are able to reward MI performance with sensory real-time feedback and consequently have been employed for motor rehabilitation following stroke.

Previous applications of BCIs for stroke rehabilitation offered promising results [5,7–12]. However, widespread application and dissemination of BCIs for stroke rehabilitation necessitates its optimization to provide clinically significant outcomes in a timely fashion. This lack of efficacy in application of BCI to restore impaired motor functions after stroke may, at least partly, be caused by suboptimal feedback provision. While some studies have investigated the effect of feedback modality on the BCI performance [13,14], to our knowledge the optimal feedback update interval (FUI) during MI remains unexplored.

Administration of paired associative stimulation (PAS) causes long-term potentiation (LTP)-like neuroplasticity in the motor cortex [15]. There is also evidence [16] that sub-threshold and high-frequency cortical stimulation delivered concurrently with intensive rehabilitation therapy resulted in behavioural gains for patients with chronic hemiparetic stroke. Passive finger flexion/extension initiates a sensory volley that arrives in the sensory cortex via the thalamo-cortical tract [17]. In turn, approximately 10 ms later, neurons in the motor cortex are disinhibited for a short period [18–20]. Therefore, this suggests that provision of recurrent sensory feedback, such as finger flexion/extension, during MI performance leads to recurrent afferent sensory signals that activate the sensory cortex and thereby disinhibit the motor cortex. There is evidence that disinhibition augments plasticity in the human motor cortex [21]. Thus, we hypothesized that shortening the FUI, i.e. provision of sensory feedback at higher frequencies, within a restorative BCI framework may provide an improved environment for the occurrence of neuroplasticity following stroke. Our prior work [22] and other groups' studies [5,14] suggest suitability of proprioceptive feedback for restorative BCIs. Therefore, in this study we used proprioceptive feedback and investigated whether shortening FUI during neurofeedback training affects motor performance following stroke.

To study the effect of neurofeedback training with shorter than usual FUIs on motor function changes after stroke, we adopted a single-case proof-of-principle study design. The adopted FUIs in the previous studies on application of BCI for stroke rehabilitation provided real-time proprioceptive feedback in the 200–300 ms range [5,11]. Therefore, we selected FUIs less than 100 ms, which are at least two times shorter than FUIs adopted in earlier studies [5,11], to detect a potential effect of FUI shortening. The action research arm test (ARAT) [23] was used as our primary outcome measure. We also used rest and active motor evoked potentials (MEPs) as well as maximum voluntary contraction (MVC) as our secondary neurophysiological tests.

## 2. Methods

### 2.1. Ethics, subjects and inclusion criteria

The study was approved by the Human Research Ethics Committee of the University of Adelaide. As a proof-of-principle study, it was planned to recruit one stroke patient. The prospective participant had to fulfil the following inclusion criteria: (i) being at least six months following stroke and in a stable condition; (ii) having impaired motor capabilities in their affected arm determined by an ARAT score less than 54 out of 57; (iii) having intact cognitive functions determined by the mini-mental state examination (MMSE) score [24] to be more than 26 out of 30; (iv) being independently mobile—with or without a walking aid; (v) not having any transcranial magnetic stimulation (TMS) contra-indications—by screening them using TMS adult safety screen (TASS) questionnaire [25]; (vi) not having excessive tone in their arm and hand muscles determined by the modified Ashworth test score [26] to be less than 3 out of 4; (vii) having the ability to perform vivid MI—by screening their accuracy in running an MI-based BCI system to be more than 70%; (viii) having an intact sense of proprioception—by screening their blind judgement of comparing size of seven polystyrene balls [27] with more than 90% accuracy. We screened three stroke patients and included a 65-year-old male. The participant had the following characteristics as tested by inclusion/exclusion criteria: (i) time after stroke: 3.75 years; (ii) ARAT Score: 34; (iii) MMSE score: 30; (iv) mobility: fully mobile; (v) TMS contra-indicator: Nil; (vi) modified Ashworth Score: 2; (vii) BCI accuracy: 75%; (viii) proprioception accuracy: 95%. Informed consent was obtained from all participants.

### 2.2. Study design

In this study, we investigate: (i) whether and how neurofeedback training with FUIs less than 100 ms affects the motor performance and (ii) in the case of observing any potential effect of these shorter than usual FUI values on behavioural and/or neurophysiological measures, how long the impacts last. Therefore, we implemented a specific study that not only recorded the performance measures during neurofeedback training sessions, but also measured the indices up to five weeks after BCI training interventions. To fulfil the aforementioned goals, a proof-of-principle study with an ABABCC set-up [28] was designed. The selected participant took part in a number of performance index measurements (PIMs) including functional scores and neurophysiological measures (see below) in non-intervention weeks (A), intervention weeks (B) and follow-up weeks (C). In non-intervention weeks, only the PIMs were done on Monday, Wednesday and Friday. In intervention weeks, on every weekday a BCI training session was performed followed by measuring indices on Monday, Wednesday and Friday. Then, in the follow-up weeks, performance indices were measured once per week, to investigate how long potential changes last, one and five weeks after the last neurofeedback training session (weeks 5 and 9). The design schedule is presented in table 1.

**Table 1.** The study was of nine weeks duration and was set as ABABCC. In A weeks (weeks 1 and 3), only performance measures were recorded three times per week. In B weeks (weeks 2 and 4), in addition to recording performance measures three times per week, five neurofeedback sessions were carried out where the FUI values for each session are shown in braces. In C weeks (weeks 5 and 9), only one recording of performance measures was performed. During weeks 6–8, no recording sessions were performed (IM: index measurement, BCI: neurofeedback training session).

	Monday	Tuesday	Wednesday	Thursday	Friday
week 1 (A)	IM	—	IM	—	IM
week 2 (B)	BCI(96) + IM	BCI(48)	BCI(48) + IM	BCI(16)	BCI(24) + IM
week 3 (A)	IM	—	IM	—	IM
week 4 (B)	BCI(16) + IM	BCI(24)	BCI(48) + IM	BCI(96)	BCI(48) + IM
week 5 (C)	—	—	—	—	IM
week 9 (C)	—	—	—	—	IM

### 2.3. Neurofeedback training set-up

#### 2.3.1. Brain–computer interface set-up: amplifier, orthoses and software

We used a 72 channel Refa TMSi EXG amplifier, containing 64 unipolar and eight bipolar channels and a 64 channel Waveguard EEG cap, for data acquisition. According to the results of the participant's screening session, an optimum frequency of 15 Hz with a large Laplacian configuration of EEG channels centred on CP4 channel produced the highest coefficient of determination [29] for the left hand MI versus relaxation trials. Note that the optimal frequency and channels were selected according to the data recorded during the screening session. For further details on the algorithm the reader is referred to [13]. To cope with real-time constraints of the BCI system with very short FUIs such as 16 ms, only five out of 64 EEG channels (FC4, CPz, CP4, PO4 and TP8) were used to record EEG signals during training sessions. The AFz channel was used as the ground channel. Also, one bipolar channel was used to record EMG activity from the extensor digitorum communis (EDC) muscle to monitor a potential voluntary finger extension during MI. The impedance between the scalp and recording electrodes were kept below 10 kΩ. The amplifier uses a built-in common average referencing algorithm where any electrode with high impedance is excluded from common average reference calculation. The sampling frequency was set to 1000 Hz. To remove DC offset and non-related high-frequency elements, a band-pass filter with corner frequencies set to 0.1 and 48 Hz was also applied.

The BCI2000 [30] was adopted as the software platform where we customized it to supply auditory commands and concurrently update servomotors' position throughout the feedback section of each trial.

**Figure 1.**
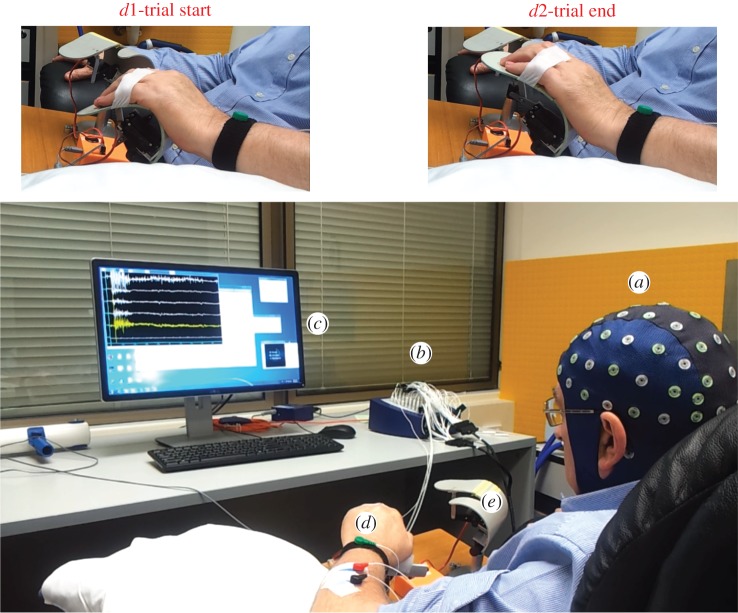
This figure illustrates the set-up of the neurofeedback training sessions. (*a*) The EEG cap records EEG signals. (*b*) The Refa EXG amplifier that receives and amplifies the EEG and EMG signals and then sends them to a PC for processing and screening. (*c*) The PC monitor that screens the EEG and EMG signals for the study instructor. (*d*) The left orthosis which is hidden under the participant's left hand and provides proprioceptive feedback during MI. (*d*1) and (*d*2) present side views of the left orthosis at the start and the end of each MI trial. (*e*) The free running orthosis that provides visual feedback during relaxation.

To provide proprioceptive feedback, we designed and fabricated two orthoses (one for each hand) to passively extend four fingers. The left orthosis was associated with the participant's affected (left) hand to provided proprioceptive feedback during MI. The right orthosis, which was not involved with the patient's hand, provide visual feedback via observation of the orthosis extension during relaxation trials. Each orthosis position was controlled via a servomotor (Blue Bird BMS-630). A Micro Maestro servo controller module translated BCI2000 commands and accordingly operated the servomotors. Figure 1 illustrates the set-up for the BCI training session.

#### 2.3.2. Time course of training sessions

**Figure 2.**
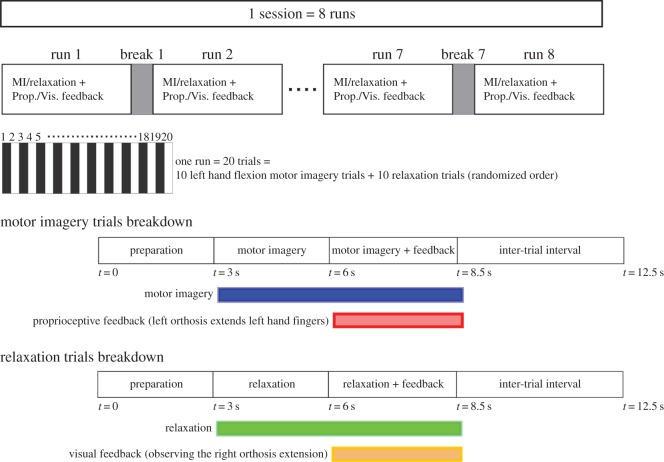
This figure illustrates the time course of each neurofeedback training session. Each session encompasses eight runs, where each run includes 20 trials. Each trial starts with a preparation cue at *t*=0 s, followed by another command at *t*=3 s that guides the participant to perform relaxation or MI of left-hand finger extension. After 3 s of MI/relaxation performance, feedback provision starts and becomes updated recurrently every 16/24/48/96 ms according to the randomized and predetermined FUI value for each session. At *t*=8.5 s, the trial finishes and after a 4 s inter-trial interval, the next trial starts.

Each session included eight runs, where each run comprised 20 trials with 10 left-hand MI and 10 relaxation trials, ordered randomly. Each trial started with an auditory cue at *t*=0 s, followed by another auditory command at *t*=3 s, which instructed the patient to perform relaxation or MI of left-hand finger extension. After 3 s of MI/relaxation performance, feedback provision started and was updated every 16/24/48/96 ms according to the randomized and predetermined FUI value for each session (see below). At *t*=8.5 s, the trial finished and after a 4 s inter-trial interval, the next trial started. Figure 2 demonstrates the time course of neurofeedback training sessions.

#### 2.3.3. Feedback update interval value selection and randomization

As discussed in the Introduction, the aim of this study was to investigate how shortening FUI duration impacts neurofeedback training for stroke patients. To highlight any potential effect, the FUIs were determined to be at least two times shorter than previously adopted values, i.e. less than 100 ms. In the meantime, to remove a potential bias of any specific FUI value on the study's results, we decided to select a number of FUIs within the 0–100 ms range and randomly assign them for each training session. Owing to the specifications of the EEG amplifier's firmware, the FUIs could be increased with 8 ms steps that dictated the largest FUI in the 0–100 ms range to be 96 ms. Thus, we decided to choose FUIs to be logarithmically equidistant, which determines their values to be 12, 24, 48 and 96 ms. However, the shortest technically possible FUI with our set-up was 16 ms. Therefore, the four chosen FUIs were as follows: 16, 24 (16×1.5), 48 (16×3) and 96 (16×6) ms. The chosen value for FUI at each training session was randomly chosen among the mentioned four values. Note that each training week comprised five training sessions while we only had four FUIs. Therefore, the repeated FUI during randomization was selected as 48 ms as it is the closest value among FUIs to their average value. Accordingly, in each training week, 16, 24 and 96 ms FUIs were used only once, while the 48 ms FUI was adopted twice.

#### 2.3.4. Signal processing

A 16th-order autoregressive model was built according to the spectral power of the most recent 500 ms time window of EEG signals. The coefficients of the autoregressive models were then used to classify whether an event-related (de)synchronization (ERD/ERS) at 15 Hz (the optimal frequency for the subject) occurred. At each FUI, if an ERD was detected, the left orthosis extended the left-hand four fingers to a fraction of a degree and rewarded MI with proprioceptive feedback. For relaxation trials, however, it was an ERS that extended the right orthosis to a fraction of a degree and rewarded relaxation trials with visual feedback through observation of the right orthosis extension.

### 2.4. Performance measures

Functional changes were monitored using the ARAT [23]. We also used rest and active MEP as well as MVC as our secondary neurophysiological tests.

#### 2.4.1. Action research arm test

The ARAT [23] is a commonly adopted and reliable measure of the upper limb function following hemiplegia. It supplies an accurate measure for the upper limb through screening functions such as grip, grasp, pinch and gross arm movement. In this study, the ARAT was employed as the primary outcome measure to investigate the effect of neurofeedback training on the recovery of the affected arm following stroke.

#### 2.4.2. Maximum voluntary contraction

To investigate how BCI training sessions affect the participant's volitional contraction of finger extensor muscles, we adopted the MVC measure [31]. The participant sat in an armchair while his left arm was placed on the armrest. An auditory command of ‘GET READY’ signalled the participant to be prepared for recording. Then, a ‘START’ auditory command, instructed him to extend his left-hand fingers with maximum power while an experienced physiotherapist was holding his hand. After 2.5 s of recording, a ‘STOP’ auditory command cued the end of the trial. Each MVC recording session encompassed three trials with 10 s inter-trial intervals. The root mean square of the EMG activity recorded from the finger extensor muscles (surface recording electrodes) for each of the three trials was calculated and then averaged, to determine the MVC measure for each session.

#### 2.4.3. Motor evoked potentials

Additionally, MEPs were recorded from EDC muscle in both rest and active conditions through application of TMS. The TMS was applied over the lesioned M1 at the optimal hotspot for evoking MEPs in EDC muscle. We used a Magstim 200 (The Magstim Co., Dyfed, UK) machine with a figure-of-eight D70^2^ coil. The coil was positioned at approximately 45^°^ to the medial line with its handle pointing towards the back of the head. The induced current in the brain with this orientation of the coil flowed in a posterior to anterior direction, which is optimal to activate the hand representation area in the motor cortex [32].

#### 2.4.4. Recording rest motor evoked potentials

To record rest MEPs, we used a 72 channel Refa TMSi EXG amplifier and used one of its bipolar channels to record EMG of the finger extensor muscles of the participants' affected, i.e. left, forearm. We used disposable electromyogram (EMG) snap electrodes with a belly-tendon set-up to record MEPs. To enable a communication between the Magstim 200 machine and the EMG amplifier, we used a custom-built Matlab program that every 8±10% s (7.2–8.8 s), sent a trigger pulse through an RS-232 communication channel to both the Magstim 200 machine and the digital input of the EMG amplifier. The recording of both the digital input pulse and the EMG signal were performed using a customized version of the Polybench software provided by TMSi. To find the hotspot, first the best point over the right motor cortex that evoked the largest MEP in the finger extensor muscles was found and marked using marker pens. Next, the intensity was increased in a number of steps and the rest motor threshold was defined as the minimal intensity with which at least 5 out of 10 consecutive MEPs had peak to peak amplitudes of larger than 50 μ*V*. The rest MEPs were then recorded at 120% of the rest motor threshold and the average peak to peak amplitude of 15 consecutive MEPs was defined as the rest MEP at each recording session.

#### 2.4.5. Recording active motor evoked potentials

**Figure 3.**
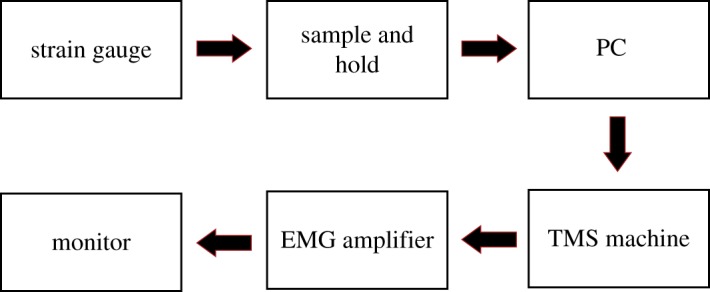
This figure illustrates building blocks of the fabricated manipulator to record active MEPs. First, the applied finger extension force is measured by a strain gauge. Then, the measured voltage is processed in a sample and hold block. Next, a PC processes the measured force and if detected to be in the desirable range i.e. 150–160 g, a trigger command is sent to the Magstim 200 machine that stimulates the brain. The Magstim machine also sends a trigger signal to an EMG amplifier to start recording. The recorded signal is then shown on a monitor.

To record active MEPs, we designed and fabricated application-specific hardware that measured the finger extension force and sent it to the PC for further processing. As soon as the applied force fell in the range of 150–160 g, a trigger command was sent to the Magstim 200 machine that stimulated the brain. Figure 3 illustrates different building blocks of the fabricated manipulator. To define the active motor threshold, the intensity of the stimulation was increased step-wise and MEPs were recorded. The minimal intensity that elicited at least 5 out of 10 MEPs larger than 200 μ*V* was defined as the active motor threshold. Then, the active MEP was defined by averaging 15 consecutive MEPs, while the participant's left-hand fingers were applying 150–160 g of force, at an intensity of 120% of the active motor threshold. Note that in weeks 2 and 4, which included neurofeedback training, TMS applications were applied after BCI training sessions to remove a potential effect of TMS application on BCI training.

## 3. Results

### 3.1. Action research arm test scores

**Figure 4.**
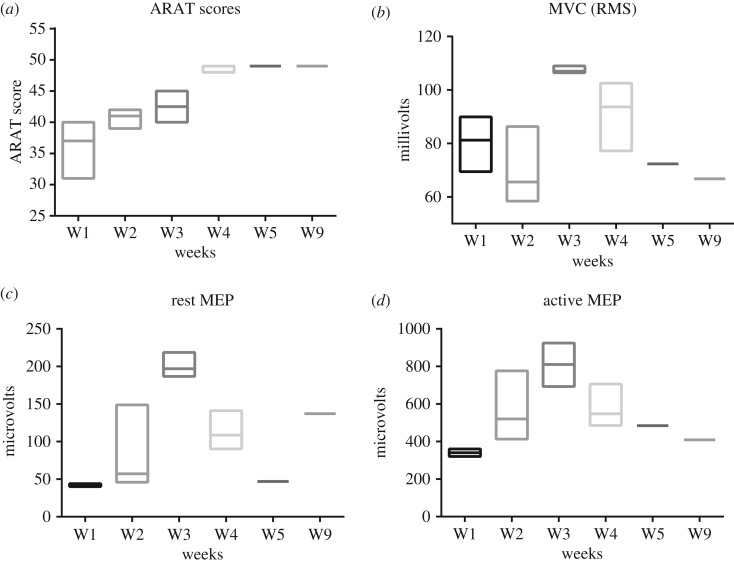
Panel (*a*) illustrates the average and standard deviation of ARAT scores along weeks 1–9 where it increases through weeks 2–5 and then plateaus. Panel (*b*) shows the trend of MVC scores where it decreases in week 2 and then increases in week 3 and finally shows a decremental trend along weeks 4–9. Panel (*c*) depicts the trend of rest MEP peak to peak amplitudes where it shows increment along weeks 2–3, followed by decrement along weeks 4–5 and finally increase in week 9. Panel (*d*) presents the trend of active MEP peak to peak amplitudes that shows an increasing trend over weeks 2–3 and then is decreased along weeks 4–9 (ARAT: arm research action test, MVC: maximum voluntary contraction, MEP: motor evoked potential).

In the first week of BCI training (week 2), the average ARAT score was 40.7, which compared to its baseline value in week 1 (36), showed a 13% increase. The ARAT scores reached 42.5 in week 3 (no BCI training) and showed an 18% increase compared to the reference ARAT score of 36. The average ARAT score in week 4, in which another round of BCI training took place, was 48 and revealed a 34% increase compared to the baseline value. In week 5, the ARAT score revealed a subtle increase of less than 2% and reached 49. The increasing trend of the ARAT scores changed after week 5, and in week 9 its value plateaued at 49. Overall, the ARAT scores revealed a 36% increase over weeks 1–5 and then plateaued at week 9, where the highest increments occurred in the weeks with BCI training (weeks 2 and 4). Figure 4*a* demonstrates the weekly averages and standard deviations of the ARAT scores.

### 3.2. Maximum voluntary contraction

MVC was found to be 80 mV in the week 1 (considered as reference value). In week 2, however, the MVC decreased by 16% and was measured at 68 mV. In week 3, the decreasing trend of the MVC changed and, with a 34% increase compared with its reference value, reached 108 mV. In week 4, even though it dropped to 91 mV and its value became smaller than that of week 3, it still remained 14% higher than the baseline. In weeks 5 and 9, it went again below baseline and reached 72 mV and 67 mV, respectively. Altogether, the MVC scores were only above the baseline value in weeks 3–4. Figure 4*b* summarizes the MVC values across weeks 1–9.

### 3.3. Rest motor evoked potentials

According to the threshold of 50 μV for any feasible rest MEP, in week 1 the rest MEP was sub-threshold (43 μV). However, in the second week the rest MEP became larger than the 50 μV threshold (54 μV). Then, in week 3 it increased again and became 203 μV. In week 4, it dropped to 113 μV. The decremental trend continued in week 5 where the rest MEP was again sub-threshold (47 μV). However, in week 9 it increased to 137 μV. Overall, the rest MEP had a rising trend over weeks 2–3 followed by a decrement along weeks 4–5, and finally ended up with an increase in week 9. Figure 4*c* depicts the rest MEP values measured along the study.

### 3.4. Active motor evoked potentials

In week 1, the baseline peak to peak value of the active MEP, where the participant was applying 150 g of force, was 340 μV. In week 2, the active MEP showed a 67% increase and reached 570 μV. In week 3, the active MEP value rose for the second time and reached 809 μV and showed a 138% increase compared with its baseline value. In week 4, the active MEP dropped to 580 μV, but it was still 70% larger than the reference. The decrement continued in week 5 and week 9 where the active MEPs were measured at 484 and 408 μV, though they were still above the baseline level by 42 and 20%, respectively. To sum up, active MEPs had an increasing trend along weeks 2–3, followed by a decreasing trend across weeks 4–9. Figure 4*d* shows the active MEPs trend along the study course.

## 4. Discussion

The main finding of this single-case study is that neurofeedback training with FUIs chosen within the 16–96 ms range may potentially have a constructive impact on the motor behaviour following a stroke. The mentioned possibility is supported by increase in the ARAT scores by 13 points (36%), which was achieved after 10 sessions of neurofeedback training. Note that any improvement in the ARAT score larger than 10% of the maximum score of 57, i.e. 5.7 is considered as a clinically significant improvement [33]. Thus, it appears that the amount of improvement in the ARAT score in the present study is clinically significant.

### 4.1. The feedback update interval shortening

Other studies on application of restorative BCIs for motor rehabilitation following stroke with real-time proprioceptive feedback adopted longer values for the FUI. Gomez-Rodriguez *et al.* [5] provided real-time proprioceptive feedback every 300 ms [5], whereas in the design of Ramos-Murguialday *et al.* [11], it was provided every 200 ms [11]. Note that studies such as Foldes *et al.* [34] adopted FUIs less than 100 ms (76 ms); however, their results are not relevant to the findings of this study, as they used visual feedback. FUI values in the current study (16, 24, 48 or 96 ms) were at least two times shorter than previous studies which adopted proprioceptive feedback. Considering the disinhibitory effect of sensory feedback on the motor cortex that lasts for a few milliseconds [18–20], the adopted shorter FUIs (i.e. more frequent sensory feedback) may have lengthened the disinhibition periods in the motor cortex. It has been reported that disinhibition through decreasing GABA-related cortical inhibition as a result of ischaemic nerve block enhances activity-dependent plasticity in people with stroke [35]. There is also evidence that disinhibition augments plasticity in the human motor cortex [21]. Therefore, these longer periods of disinhibition may have enhanced the occurrence of plasticity in the motor cortex that was manifested in the observed increment in the ARAT scores.

### 4.2. Early and late phases of learning

The second implication of the current study is that short- and long-term impacts of BCI training are reflected distinctively through behavioural measures such as ARAT, and physiological measures such as rest and active MEP amplitudes, and MVC. All measures showed an increasing trend (despite some small deviations), both during (week 2) and after earlier sessions of BCI training (week 3). However, the ARAT scores increased, whereas rest/active MEP amplitudes and MVC values decreased both during (week 4) and after (week 5) later sessions of neurofeedback training. Karabanov *et al.* [36] have explored connectivity between the posterior parietal cortex (PPC), which is thought to play a critical role in sensory information processing, and the primary motor cortex (M1) in the early and late phases of motor learning [36]. They reported that while connectivity between M1 and PPC increases in the early phases of motor learning, it degrades during the late phases of motor learning. Presuming that acquiring MI skills takes longer than learning motor skills, we can find congruency between the findings of Karabanov *et al.* and the observations of this study: during week 2 of this study, which can be considered as the early phase of MI training, the connectivity between M1 and PPC may have increased and thereby increased rest and active MEPs. However, in week 4, which can be regarded as a late phase of MI training, it had potentially become automatized [37]. Therefore, the decreased rest/active MEPs may have been caused by decreased connectivity between M1-PPC in week 4.

### 4.3. Implicit and explicit phases of learning

Pascual-Leone *et al.* [38] reported that during implicit motor learning, participant reaction time decreased and the cortical map of muscles involved in the learning task extended [38]. However, when explicit knowledge of the motor learning task was achieved, the reaction time stopped decreasing and the cortical representation of the related area shrank. The observation we had in this study was at least to some extent similar to the results of Pascual-Leone *et al*. In their study, the reaction time was a behavioural measure that resembles our adopted ARAT score. They also used the paired-pulse TMS as their neurophysiological index, which is similar to the active MEP measure of this study. BCI training in week 2 (first week of BCI training) can also be considered as the implicit phase of the training of the MI task. The implicit phase of training in week 2 was manifested in increasing ARAT scores, and active MEP values, similar to reduction in reaction time and increase in MEP values in the study of Pascual-Leone *et al.* Furthermore, during week 3, where no training took place, we observed that all measures rose, which may reflect the consolidation of implicit motor learning. However, after week 4, when the second round of BCI training occurred, the motor learning may have switched to its automatized (explicit) phase similar to a well-learned motor skill [37]. Thus, congruent with findings of Pascual-Leone and colleagues, our clinical measure (ARAT score) plateaued in week 5, whereas all other physiological indices such as MVC and MEPs reduced. Thus, these observations may reflect the shrinkage of the cortical representation of the related area to the trained MI task.

Altogether, our observations suggest that (i) similar to learning a new skill, in early phases of MI training the cortical representation of the related area extends and (ii) after consolidation of MI task, the extended cortical representation of the task shrinks while the MI skill remains intact.

## 5. Limitation of the study

Reported results of this study are obtained from a single-case proof-of-principle study design and, therefore, it was not possible to be statistically analysed. Thus, the reported findings need to be validated with larger studies in which sufficient numbers of participants are recruited and divided into target and control groups with different adopted FUI ranges.

## 6. Conclusion

In this single-case proof-of-principle study, we used a restorative BCI to investigate (i) whether adopting FUIs within the 16–96 ms range for neurofeedback training following stroke affects behavioural and clinical measures of motor rehabilitation; (ii) the duration of any potential training-induced changes in behavioural or neurophysiological measures. We observed that (i) the adopted shorter than usual FUI values for restorative BCIs may potentially enhance the motor performance following stroke; (ii) following earlier phases of BCI training, all behavioural and physiological measures increased (week 3); (iii) after the later phases of neurofeedback training, the behavioural index (ARAT) plateaued, while the physiological measures decreased (week 5). The observed trend may reflect the impact of early (or implicit) and late (or explicit) phases of MI training. Further studies that investigate the mentioned factors (early and late phases of MI training, and duration of FUI) separately with larger sample sizes are required to determine whether and to what extent each factor affects the motor rehabilitation via neurofeedback training following stroke.

## Data accessibility

Our data are deposited at Dryad: http://dx.doi.org/10.5061/dryad.97bt8 [39].
